# Genome Sequence of Mycobacterium abscessus Phage phiT45-1

**DOI:** 10.1128/MRA.00155-21

**Published:** 2021-03-11

**Authors:** Elizabeth D. Amarh, Christian H. Gauthier, Rebekah M. Dedrick, Rebecca A. Garlena, Daniel A. Russell, Deborah Jacobs-Sera, Kira M. Zack, Graham F. Hatfull

**Affiliations:** a Department of Biological Sciences, University of Pittsburgh, Pittsburgh, Pennsylvania, USA; DOE Joint Genome Institute

## Abstract

Mycobacteriophage phiT45-1 is a newly isolated bacteriophage spontaneously released from Mycobacterium abscessus strain Taiwan-45 that lytically infects M. abscessus strain BWH-C; phiT45-1 also infects M. abscessus ATCC 19977 but not Mycobacterium smegmatis. Phage phiT45-1 has a 43,407-bp genome and carries a polymorphic toxin-immunity cassette associated with type VII secretion systems.

## Abstract (precis)

Mycobacteriophage phiT45-1 is a newly isolated bacteriophage spontaneously released from Mycobacterium abscessus strain Taiwan-45 that lytically infects M. abscessus strain BWH-C; phiT45-1 also infects M. abscessus ATCC 1997 but not Mycobacterium smegmatis. Phage phiT45-1 has a 43,407-bp genome and carries a polymorphic toxin-immunity cassette associated with type VII secretion systems.

## ANNOUNCEMENT

Nontuberculous mycobacteria (NTM) are mycobacterial species that do not cause tuberculosis or leprosy (1). Among the many NTM pathogens, Mycobacterium abscessus is often antibiotic resistant and refractory to treatment. M. abscessus infections are frequent among cystic fibrosis patients and those with bronchiectasis and can disseminate in immunosuppressed patients (2, 3). The robust nature of M. abscessus contributes to the prevalence of latent infections and the evolution of multidrug-resistant (MDR) strains (1). The rise of antibiotic resistance in M. abscessus cases has prompted consideration of mycobacteriophages—viruses that infect mycobacteria—as a therapeutic alternative (4).

**FIG 1:**
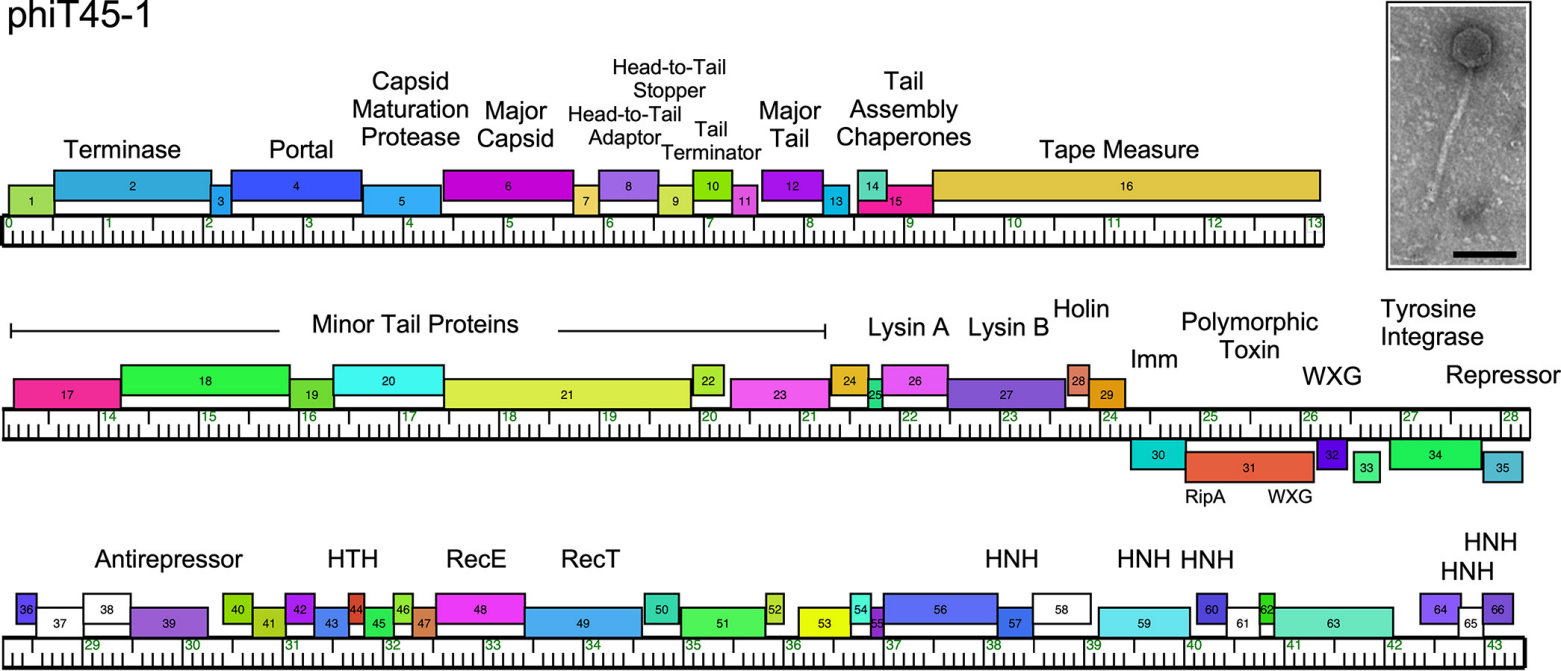
Genome organization of phage phiT45-1. The viral genome of phiT45-1 is represented as a horizontal bar with vertical markers every kilobase pair. Genes are represented by colored boxes above and below the genome, indicating rightward and leftward transcription, respectively; white boxes represent orphams, genes with no close relatives in this data set. Gene numbers are shown in each box. Genes are colored according to their phamily assignments, determined using Phamerator (11) with the database Abscessus_phage_and_prophage v3. Putative gene functions are indicated. The inset shows an electron micrograph of phiT45-1; the scale marker is 100 nm.

It is not uncommon for strains of M. abscessus to contain prophages (5), and spontaneous release of phage particles from such strains has been previously described (6). Phage phiT45-1 was isolated by plating culture supernatant from M. abscessus Taiwan-45 onto a lawn of M. abscessus strain BWH-C (both provided by Chidiebere Akusobi and Eric Rubin) on solid medium at 37°C using standard methods (7). Phage were picked from infected areas, plaque purified, and amplified on BWH-C (7), followed by DNA extraction using the Wizard DNA cleanup system (catalog no. A7280; Promega, Madison, WI). Sequencing libraries were prepared from genomic DNA by using a NEBNext Ultra II FS kit with dual-indexed barcoding. Forty-eight libraries were pooled and run on the Illumina MiSeq platform, yielding 192,000 single-end 150-bp reads and 500-fold coverage of the genome. The raw sequence reads were assembled using Newbler v2.9 with default settings, yielding a single phage contig of 43,407 bp with 65% G+C content. The contig was assessed for completeness, accuracy, and phage genomic termini determination using Consed v29 as previously described (8); the viral genome sequence has defined ends with 10-base 3′ single-strand extensions. Protein-coding genes were identified using GeneMarkS v4.30 (9), Glimmer v3.02 (10), the Phamerator database Abscessus_phage_and_prophage v3 (11, 12), and DNA Master v5.23.5 (http://cobamide2.bio.pitt.edu) (Fig. 1). Putative functions were assigned to 52% of the 66 protein-coding genes using BLAST (13) and HHpred (14, 15). No tRNA genes were identified by ARAGORN v1.2.41 (16). All tools were run with default parameters unless otherwise stated.

Phage phiT45-1 does not have overall similarity (all BLASTN bit scores of <190) to phages isolated on M. smegmatis (17), although its portal, capsid maturation protease, and capsid proteins (*4*, *5*, and *6*, respectively; Fig. 1) share >60% amino acid identity with cluster N mycobacteriophages, which have genome sequence lengths similar to that of phiT45-1 (18, 19); like cluster N phages, phiT45-1 also has a siphoviral morphology (family *Siphoviridae*) (Fig. 1). Early lytic genes include a RecET-like recombination system (*48* and *49*) and several predicted HNH endonucleases (*57*, *59*, *60*, *65*, and *66*; Fig. 1). The presence of a tyrosine integrase (*34*) and immunity repressor (*35*) is consistent with phiT45-1 being temperate. Interestingly, phiT45-1 codes for a polymorphic toxin (PT) cassette, including an immunity protein (*30*), a polymorphic toxin (*31*) with RipA-like and WXG-100 domains (20, 21), and a WXG-100 protein (*32*) (22), situated close to the integrase and repressor genes, and likely lysogenically expressed; phiT45-1 gp31 and gp32 are presumably exported via a type VII secretion system. A similar PT system has been reported for M. abscessus phage phiT46-1 (6).

### Data availability.

Phage phiT45-1 is available at GenBank under accession no. MW570842 and BioProject accession no. PRJNA488469. The sequencing reads are available in the SRA under accession no. SRX10050651.

## References

[1] Johansen MD, Herrmann J-L, Kremer L. 2020. Non-tuberculous mycobacteria and the rise of Mycobacterium abscessus. Nat Rev Microbiol 18:392–407. doi:10.1038/s41579-020-0331-1. 32086501

[2] Hasan NA, Davidson RM, Epperson LE, Kammlade SM, Rodger RR, Levin AR, Sherwood A, Sagel SD, Martiniano SL, Daley CL, Salfinger M, Nick JA, Strong M. 2019. Population genomics of nontuberculous mycobacteria recovered from United States cystic fibrosis patients. bioRxiv 10.1101/663559.

[3] Kavaliunaite E, Harris KA, Aurora P, Dixon G, Shingadia D, Muthialu N, Spencer H. 2020. Outcome according to subspecies following lung transplantation in cystic fibrosis pediatric patients infected with Mycobacterium abscessus. Transpl Infect Dis 22:e13274. doi:10.1111/tid.13274. 32129923

[4] Dedrick RM, Guerrero Bustamante CA, Garlena RA, Russell DA, Ford K, Harris K, Gilmour KC, Soothill J, Jacobs-Sera D, Schooley RT, Hatfull GF, Spencer H. 2019. Engineered bacteriophages for treatment of a patient with a disseminated drug-resistant *Mycobacterium abscessus*. Nat Med 25:730–733. doi:10.1038/s41591-019-0437-z. 31068712 PMC6557439

[5] Glickman C, Kammlade SM, Hasan NA, Epperson LE, Davidson RM, Strong M. 2020. Characterization of integrated prophages within diverse species of clinical nontuberculous mycobacteria. Virol J 17:124. doi:10.1186/s12985-020-01394-y. 32807206 PMC7433156

[6] Amarh ED, Dedrick RM, Garlena RA, Russell DA, Jacobs-Sera D, Hatfull GF. 2021. Genome sequence of Mycobacterium abscessus phage phiT46-1. Microbiol Resour Announc 10:e01421-20. doi:10.1128/MRA.01421-20. 33446600 PMC7849713

[7] Sarkis GJ, Hatfull GF. 1998. Mycobacteriophages. Methods Mol Biol 101:145–173. doi:10.1385/0-89603-471-2:145. 9921476

[8] Russell DA. 2018. Sequencing, assembling, and finishing complete bacteriophage genomes. Methods Mol Biol 1681:109–125. doi:10.1007/978-1-4939-7343-9_9. 29134591

[9] Besemer J, Borodovsky M. 2005. GeneMark: Web software for gene finding in prokaryotes, eukaryotes and viruses. Nucleic Acids Res 33:W451–W454. doi:10.1093/nar/gki487. 15980510 PMC1160247

[10] Delcher AL, Bratke KA, Powers EC, Salzberg SL. 2007. Identifying bacterial genes and endosymbiont DNA with Glimmer. Bioinformatics 23:673–679. doi:10.1093/bioinformatics/btm009. 17237039 PMC2387122

[11] Cresawn SG, Bogel M, Day N, Jacobs-Sera D, Hendrix RW, Hatfull GF. 2011. Phamerator: a bioinformatic tool for comparative bacteriophage genomics. BMC Bioinformatics 12:395. doi:10.1186/1471-2105-12-395. 21991981 PMC3233612

[12] Mavrich TN, Gauthier C, Abad L, Bowman CA, Cresawn SG, Hatfull GF. 2020. pdm_utils: a SEA-PHAGES MySQL phage database management toolkit. Bioinformatics: btaa983. doi:10.1093/bioinformatics/btaa983. 33226064 PMC8388035

[13] Altschul SF, Gish W, Miller W, Myers EW, Lipman DJ. 1990. Basic local alignment search tool. J Mol Biol 215:403–410. doi:10.1016/S0022-2836(05)80360-2. 2231712

[14] Remmert M, Biegert A, Hauser A, Soding J. 2011. HHblits: lightning-fast iterative protein sequence searching by HMM-HMM alignment. Nat Methods 9:173–175. doi:10.1038/nmeth.1818. 22198341

[15] Soding J, Biegert A, Lupas AN. 2005. The HHpred interactive server for protein homology detection and structure prediction. Nucleic Acids Res 33:W244–W248. doi:10.1093/nar/gki408. 15980461 PMC1160169

[16] Laslett D, Canback B. 2004. ARAGORN, a program to detect tRNA genes and tmRNA genes in nucleotide sequences. Nucleic Acids Res 32:11–16. doi:10.1093/nar/gkh152. 14704338 PMC373265

[17] Hatfull GF. 2020. Actinobacteriophages: genomics, dynamics, and applications. Annu Rev Virol 7:37–61. doi:10.1146/annurev-virology-122019-070009. 32991269 PMC8010332

[18] Dedrick RM, Jacobs-Sera D, Guerrero Bustamante CA, Garlena RA, Mavrich TN, Pope WH, Cervantes Reyes JC, Russell DA, Adair T, Alvey R, Bonilla JA, Bricker JS, Brown BR, Byrnes D, Cresawn SG, Davis WB, Dickson LA, Edgington NP, Findley AM, Golebiewska U, Grose JH, Hayes CF, Hughes LE, Hutchison KW, Isern S, Johnson AA, Kenna MA, Klyczek KK, Mageeney CM, Michael SF, Molloy SD, Montgomery MT, Neitzel J, Page ST, Pizzorno MC, Poxleitner MK, Rinehart CA, Robinson CJ, Rubin MR, Teyim JN, Vazquez E, Ware VC, Washington J, Hatfull GF. 2017. Prophage-mediated defence against viral attack and viral counter-defence. Nat Microbiol 2:16251. doi:10.1038/nmicrobiol.2016.251. 28067906 PMC5508108

[19] Russell DA, Hatfull GF. 2017. PhagesDB: the actinobacteriophage database. Bioinformatics 33:784–786. doi:10.1093/bioinformatics/btw711. 28365761 PMC5860397

[20] Ruggiero A, Marasco D, Squeglia F, Soldini S, Pedone E, Pedone C, Berisio R. 2010. Structure and functional regulation of RipA, a mycobacterial enzyme essential for daughter cell separation. Structure 18:1184–1190. doi:10.1016/j.str.2010.06.007. 20826344

[21] Healy C, Gouzy A, Ehrt S. 2020. Peptidoglycan hydrolases RipA and Ami1 are critical for replication and persistence of Mycobacterium tuberculosis in the host. mBio 11:e03315-19. doi:10.1128/mBio.03315-19. 32127458 PMC7064781

[22] Conrad WH, Osman MM, Shanahan JK, Chu F, Takaki KK, Cameron J, Hopkinson-Woolley D, Brosch R, Ramakrishnan L. 2017. Mycobacterial ESX-1 secretion system mediates host cell lysis through bacterium contact-dependent gross membrane disruptions. Proc Natl Acad Sci U S A 114:1371–1376. doi:10.1073/pnas.1620133114. 28119503 PMC5307465

